# Gray Matter Abnormalities of Orbitofrontal Cortex and Striatum in Drug-Naïve Adult Patients With Obsessive-Compulsive Disorder

**DOI:** 10.3389/fpsyt.2021.674568

**Published:** 2021-06-08

**Authors:** Zhang Bowen, Tan Changlian, Liu Qian, Peng Wanrong, Yang Huihui, Liu Zhaoxia, Li Feng, Liu Jinyu, Zhu Xiongzhao, Zhong Mingtian

**Affiliations:** ^1^Guangdong Key Laboratory of Mental Health and Cognitive Science, Center for Studies of Psychological Application, School of Psychology, South China Normal University, Guangzhou, China; ^2^Department of Radiology, Second Xiangya Hospital, Central South University, Changsha, China; ^3^Medical Psychological Center, The Second Xiangya Hospital, Central South University, Changsha, China; ^4^Medical Psychological Institute, Central South University, Changsha, China

**Keywords:** orbitofrontal cortex, striatum, local gyrification index, lateralization index, executive function

## Abstract

**Objective:** This study examined whether obsessive-compulsive disorder (OCD) patients have gray matter abnormalities in regions related to executive function, and whether such abnormalities are associated with impaired executive function.

**Methods:** Multiple scales were administered to 27 first-episode drug-naïve OCD patients and 29 healthy controls. Comprehensive brain morphometric indicators of orbitofrontal cortex (OFC) and three striatum areas (caudate, putamen, and pallidum) were determined. Hemisphere lateralization index was calculated for each region of interest. Correlations between lateralization index and psychological variables were examined in OCD group.

**Results:** The OCD group had greater local gyrification index for the right OFC and greater gray matter volumes of the bilateral putamen and left pallidum than healthy controls. They also had weaker left hemisphere superiority for local gyrification index of the OFC and gray matter volume of the putamen, but stronger left hemisphere superiority for gray matter volume of the pallidum. Patients' lateralization index for local gyrification index of the OFC correlated negatively with Yale-Brown Obsessive Compulsive Scale and Dysexecutive Questionnaire scores, respectively.

**Conclusion:** Structural abnormalities of the bilateral putamen, left pallidum, and right OFC may underlie OCD pathology. Abnormal lateralization in OCD may contribute to the onset of obsessive-compulsive symptoms and impaired executive function.

## Introduction

Obsessive-compulsive disorder (OCD) is a severe and potentially disabling mental disorder that is associated with neurodevelopmental risk factors (1). Commonly, OCD patients suffer from various types of cognitive impairment (2), such as altered flexibility (3), inhibition (4), and decision-making (5, 6).

There has been a growth in research efforts focused on the pathological mechanism of OCD (1, 7, 8). High-resolution brain magnetic resonance imaging (MRI) techniques investigating the cerebral changes associated with OCD and the pathological mechanism underlying OCD have revealed structural and functional abnormalities (1, 7, 8). Functional hyperactivity in the cortical-striatal-thalamic-cortical (CSTC) pathway has been suggested to underlie the manifestations of OCD (9, 10). A previous connectionism study indicated that functional alterations in the CSTC pathway were associated with structural alterations affecting connectivity between the orbitofrontal cortex (OFC) and the striatum (11, 12). Structural MRI studies have also revealed anatomical abnormalities of the OFC (13–15), striatum (16–18), and anterior cingulate cortex (ACC) (19), thalamus, hippocampus (1), and occipital cortex (20).

The CSTC pathway, a putative pathological loop of OCD (21, 22), is a multi-synaptic neuronal circuit that connects the cortex, striatum, and thalamus. It mediates important psychological functions, including movement selection and execution, behavioral initiation, habit formation, and reward. Oscillations and synchronous activity in CSTC pathway brain regions are important for the execution of habitual actions (9). Thus, synaptic dysfunction in the CSTC loop in OCD may underlie the repetitive involuntary movements associated with the disorder (10).

Research examining OCD has been focused on the OFC and striatum, which have been related to decision-making, emotion control, and cognition (7, 15, 23). Multiple studies have found an altered gray matter volume (GMV) of the OFC in OCD patients, though the direction and location of the alteration have been variable (13, 14, 24). Christian reported OCD had an greater GMV of the left OFC than healthy controls (HCs) (14), whereas van den Heuvel found a reduced GMV of the left OFC (25). Additionally, Radua et al. reported that OCD had a smaller surface area (SA) of the right OFC (17). Fouche et al. and Shin et al. both reported reduced cortical thickness (CT) of the left OFC and right inferior frontal cortex (8, 26).

The striatum consists of the caudate nucleus, putamen, and pallidum. Various structural alterations of the striatum have been found in OCD studies (15, 21, 24, 27). Some have reported greater GMVs of the caudate nucleus in OCD (16, 17), whereas others found reduced GMVs of the caudate nucleus and putamen in OCD (18, 28) and an inverse correlation between striatal GMV and OCD symptom severity (28). A meta-analysis also indicated that adult patients with OCD had significantly larger pallidum volumes compared with controls (1).

The aforementioned inconsistencies could be due to multiple factors. Firstly, cohort heterogeneity, such as depressive comorbidity, treatment, age of onset, disease course, and symptom severity, may contribute to the disparate findings. Secondly, although there may be atypical lateralization among psychiatric patients (29), most OCD studies have not accounted for lateralization. Thirdly, noise may be introduced by different researchers employing different methodological paradigms, such as focusing on a priori regions of interest (ROIs) vs. whole brain analysis, the use of voxel-based vs. surface-based morphometric analyses, and the use of different morphometric indicators.

Commonly, structural MRI studies of OCD have examined GMV, SA, and CT. Meanwhile, there remains little information regarding cortical folding in OCD. GMV is determined by both SA and CT, parameters that are influenced by different developmental factors. Cortical SA increases with cortical folding during the later stages of fetal development and CT changes dynamically across the lifespan as a consequence of development and disease (30). The differing developmental trajectories of these properties is likely to be reflected in the morphometric indicator findings associated with them.

Most studies that have examined cortical folding in OCD have focused on the ACC (19), prefrontal cortex (31), and occipital cortex (20). Wobrock found that OCD patients had hypogyrification of the left prefrontal cortex compared with HCs and pointed out that it might be a structural correlate of OCD-related impairments in executive function (EF) (31). Rus et al. found hypogyrification of rostral middle frontal regions as well as a positive association of age of onset with average local gyrification index (LGI) of the right hemisphere, including the insula, rostral middle frontal cortex, and lateral OFC (32). Venkatasubramanian found inverse correlations between OCD patients' Yale-Brown Obsessive Compulsive Scale (Y-BOCS) compulsion and insight scores with LGI values obtained for the right OFC and left medial OFC, respectively (33). Cortical folding is an indicator of early neurodevelopment, and OCD has been associated with early neurodevelopmental abnormalities (33). Thus far, there have been few studies focused on OFC folding in OCD patients.

Lateralization of cortical and subcortical structure abnormalities have been described in some patients with neuropsychiatric diagnoses (34, 35). Although few studies have focused on brain lateralization in OCD patients directly, some studies have provided hints of possible abnormal lateralization patterns in OCD. For example, Chen found that patients with OCD showed weaker connectivity between left caudate nucleus and thalamus compared with controls, which was also correlated with the duration of OCD (22). Hu et al. found that a greater GMV of the left putamen was most prominent in samples with higher percentages of medicated adult OCD patients (27). Lazaro observed that treatment of OCD patients was associated with a greater GMV of the right striatum (24). These studies suggest that OCD may be related to left hemisphere-lateralized brain alterations.

EF impairments, such as executive inhibition, transformation, refreshment, and working memory impairments, represent the core features of OCD (23, 36, 37). Neuroimaging researches suggested that EF impairments in OCD were related to malfunction of CSTC loop (37–39), and excessive activation of the OFC and striatum might be the most prominent findings in OCD patients (37, 40, 41). The OFC is strongly involved in the process of response inhibition, planning and problem solving, therefore, its dysfunction might result in corresponding EF impairment (40–42). While the striatum collects, modulates and integrates cortical information for EF, its dysfunction might impair EF, such as inhibition and decision making (43). However, the relationship between lateralized OFC/striatum differences and the clinical manifestations of OCD, especially impaired EF, is lacking.

This study aimed to test the hypothesis that impaired executive function in OCD patients is related to gray matter abnormalities, including cortical folding and hemispherical lateralization, in regions related with executive function, with OFC and striatum as ROIs.

## Materials and Methods

### Subjects

Twenty-seven drug-naïve young OCD patients participated in this study. All patients were recruited from outpatient clinics affiliated with the Second Xiangya Hospital of Central South University in Changsha, Hunan, China. The diagnoses of OCD and axis I psychiatric comorbidities were established independently by two highly-experienced psychiatrists based on the Structured Clinical Interview for DSM-IV. The inclusion criteria were: (1) ≥18 years old; (2) ≥9 years of formal education; (3) compliance with OCD diagnostic criteria in the DSM-IV; (4) first-episode and drug-naïve status; (5) no past or current other axis I diagnosis.

Twenty-nine age-matched community population participants were recruited to form the HC group. HCs were evaluated independently by two highly-experienced psychiatrists to rule out any axis I/II psychiatric disorders based on the Structured Clinical Interview for DSM-IV. The inclusion criteria were: (1) ≥18 years old; (2) ≥9 years of formal education; (3) axis I/II psychiatric disorders ruled out.

The exclusion criteria for all participants were: (1) a history of major medical or neurological problems (e.g., hypothyroidism, seizure disorder, or brain injury); (2) MRI contraindication; (3) taking drugs that affect cognitive function or related treatment; (4) being pregnant, lactating, or preparing for pregnancy; (5) inability to cooperate with the MRI procedure.

The study was conducted in accord with the Declaration of Helsinki and approved by Ethics Committee of the Second Xiangya Hospital of Central South University. All subjects provided written informed consent after receiving a full explanation of the purpose and procedures of the study.

### Self-Report Instruments

At the time of recruitment, participants completed the Chinese versions of several psychological assessments, including the self-reported Beck Depression Inventory (BDI), the State-Trait Anxiety Inventory (STAI), and Dysexecutive Questionnaire (DEQ). Patients with OCD also completed the Y-BOCS. The BDI and STAI were used to assess depression and anxiety levels, respectively (44). The DEQ was applied to detect executive dysfunction (45). The Y-BOCS was used to assess the severity and symptom profile of obsessive-compulsive disorder (46). All of the scales used in this study have been shown to have good reliability and validity (44, 47–49), see Supplementary Material for details.

### MRI Data Acquisition

Imaging data were acquired on a Siemens Skyra 3-T magnetic resonance scanner at the Second Xiangya Hospital of Central South University. We collected three-dimensional T1-weighted magnetization-prepared rapid gradient echo sagittal images (50), with the follow parameters: 176 slices, 1,900-ms repetition time, 2.01-ms echo time, 1.00-mm slice thickness, 1.00-mm^3^ voxel size, 9° flip angle, 900-ms inversion time, 256-mm field of view, and 256 × 256 matrix (51).

### Image Processing

Image data were processed by surface-based, multi-step, and semi-automated morphometric analyses. The FreeSurfer image analysis software suite was used to generate a cortical surface representations (composed of a mesh of triangles) for measurement of GMV, SA, and CT and determination of LGI values at each vertex. Preprocessing steps included visual inspection of data for motion artifacts, transformation to Talairach space, intensity normalization for correction of magnetic field inhomogeneities, and removal of non-brain tissues. For volumetric data, FreeSurfer classified cortical structures automatically. The analysis was enhanced by reference to the ROIs defined anatomically from the 2009 Destrieux Atlas during FreeSurfer segmentation (52).

**Figure 1:**
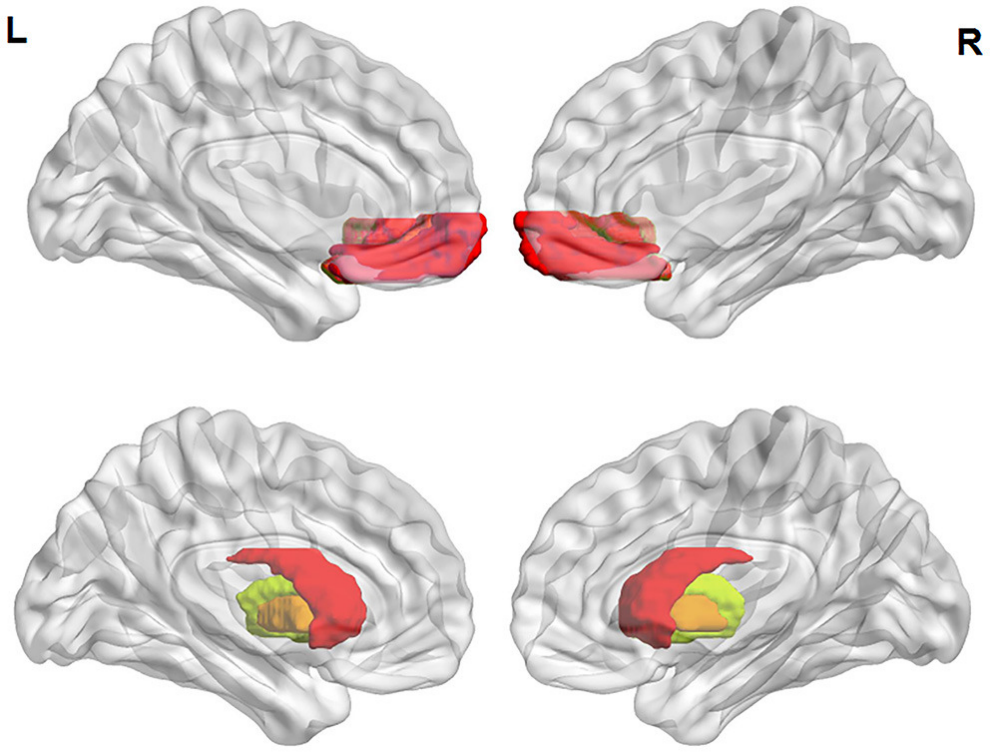
ROI locations. The ROIs defined as in the 2009 Destrieux Atlas. Pseudocolored areas show pial surface representations of the OFC (above) and volume representations of the striatum (below), wherein red shows the caudate nucleus, yellow shows the putamen, and orange shows the pallidum. ROI, region of interest; L, left; R, right.

The ROIs used in this study were the OFC and striatum (Figure 1). The following four morphometric parameters (per vertex) were extracted with FreeSurfer: GMV in cortex, SA (of pial surfaces), CT (the distance between white matter and pial surfaces), and LGI. LGI was calculated to evaluate the cortical folding pattern and utilized in the context of the FreeSurfer analysis suite. The original gyrification index was obtained by the following four steps: (1) three-dimensional reconstruction of pial cortical surface; (2) delineation of the outer hull that tightly warps the pial surface; (3) computation of LGI for each outer-surface vertex; and (4) propagation of LGI values from the outer surface mesh to the pial surface mesh to produce a cortical map of LGIs (53).

### Statistical Analyses

Surface-based group analyses were performed with FreeSurfer's general linear model tools. Prior to group comparison, each participant's data were resampled into a common anatomical space. Surface-based measurements of GMV, SA, CT, and LGI for all subjects were smoothed with 5-mm full-width at half-maximum Gaussian kernels. After defining the OFC and striatum as ROIs in volume space, we transformed each spherical ROI volume into an area (cluster) ROI in the surface space and measured the mean GMV, SA, CT, and LGI of each cluster ROI for each subject.

Differences in demographic and clinical characteristics between the OCD and HC groups were detected by two-sample *t*-tests or chi-squared test in SPSS software (version 22.0; Chicago, IL). Linear correlation of ROI variables with Y-BOCS and DEQ scores were assessed by Pearson correlation analysis. Regional GMV, SA, CT, and LGI data were subjected to analysis of repeated measurement variance with covariates (ANCOVAs), with hemisphere (left or right) as a within-subject factor, group as a between-subjects factor, and intracranial volume as a covariate. Main effects and interactions were evaluated with Greenhouse-Geisser corrected degrees of freedom and had a significance criterion of *p* < 0.05 before *post hoc* pairwise contrasts.

Laterality Index (LI) was calculated for each subject, see Supplementary Materials. Mean LI values are reported for each group with standard deviations (SDs). Shapiro-Wilk test was used to verify the normality of LI (54), which were shown in Supplementary Table 1. For the LI of putamen and pallidum were non-normal data (*p*s < 0.05), two sample *t* test and permutation test (*N* = 10,000) were applied to examine the regional LI differences between OCD group and HC group, respectively (55). Two-tailed partial correlation analyses between LI and psychological variables were performed with intracranial volume as the control variable. Effect size η^2^ and Cohen's *d* values were indicated where appropriate (56, 57).

## Results

### Demographic and Clinical Data

The characteristics of the subjects in each group are summarized in Table 1. All subjects were right handed and there were no significant group differences in age, sex, or intracranial volume (all *p* > 0.05). The OCD group had significantly higher BDI, STAI-S, STAI-T, and DEQ scores than the HC group.

**Table 1:** Demographic and psychological variables of subjects by group.

**Parameter**	**OCD (*N* = 27), Mean (SD)**	**HC (*N* = 29), Mean(SD)**	**Statistic (t^†^X^†^)**	***p^§^***	**Cohen's d**
Age, years	21.04 (5.42)	22.83 (2.22)	−1.60	0.12	-
Sex, male/female	14/13	12/17	0.62	0.43	-
Education, years	13.07 (3.13)	14.31 (3.04)	−1.55	0.12	-
Disease duration, mos.	34.11 (48.00)	-	-	-	-
**Psychometrics**					
BDI	17.48 (9.54)	6.59 (5.32)	5.23^****^	<0.001	1.41
STAI-S	54.59 (11.09)	36.66 (9.26)	6.59^****^	<0.001	1.77
STAI-T	55.74 (7.69)	39.66 (9.25)	7.05^****^	<0.001	1.89
DEQ Total	30.67 (12.91)	22.34 (11.59)	2.54^*^	0.014	0.68
Inhibition	8.70 (5.04)	6.41 (3.77)	1.94	0.058	-
Intentionality	10.33 (4.81)	7.69 (3.84)	2.28^*^	0.027	0.61
Executive memory	4.04 (2.68)	2.14 (1.66)	3.16^***^	0.003	0.85
Positive affect	4.67 (1.88)	3.41 (2.34)	2.199^*^	0.032	0.59
Negative affect	2.93 (1.71)	2.69 (1.73)	0.51	0.61	-
Y-BOCS total	30.63 (5.50)	-	-	-	-
Y-BOCS-CS	13.81 (4.57)	-	-	-	-
Y-BOCS-OS	16.81 (2.47)	-	-	-	-
Intracranial volume(ml)	1672.58 (132.41)	1605.55 (191.08)	1.52	0.14	-

*OCD, obsessive-compulsive disorder; HC, healthy control; SD, standard deviation; BDI, Beck Depression Inventory; STAI-S, Spielberger State-Trait Anxiety Inventory-State Form; STAI-T, Spielberger State-Trait Anxiety Inventory-Trait Form; DEQ, Dysexecutive Questionnaire; Y-BOCS, Yale-Brown Obsessive Compulsive Scale; Y-BOCS-CS, Y-BOCS compulsion score; Y-BOCS-OS, Y-BOCS obsession score. Data are given as mean (standard deviation) unless otherwise indicated*.

† *Independent sample t-test. ^‡^ Chi-square test*.

§ *2-tailed, significance at p = 0.05;*

* *p < 0.05;*

*** *p < 0.005;*

**** *p < 0.001*.

### Morphometrics of the OFC and Striatum

Mean regional GMV, SA, CT, and LGI values obtained for the OFC and GMV for the three substructures of the striatum are reported for the left hemisphere and right hemisphere in Table 2.

**Table 2:** Means(SDs) of gray matter structural metrics for each group in each ROI and comparison of LI values between OCD and HC.

**ROI**	**Variable**	**OCD(*****N*** **= 27)**			**HC(*****N*** **= 29)**			**Statistic (t^†^)**	***p*^†^**	**Cohen's d**
		**Left**	**Right**	**LI**	**Left**	**Right**	**LI**			
OFC	GMV	1279.56 (231.97)	1219.74 (273.44)	2.75 (11.41)	1242.28 (224.05)	1081.76 (219.19)	7.08 (9.94)	−1.513	0.136	-
	SA	292.63 (53.85)	283.89 (53.59)	1.50 (11.41)	295.24 (47.38)	264.41 (51.72)	5.68 (10.37)	−1.438	0.156	-
	CT	3.01 (0.13)	3.02 (0.24)	−0.05 (4.81)	2.93 (0.22)	2.96 (0.23)	−0.52 (3.77)	−0.493	0.624	-
	LGI	2.46 (0.16)	2.47 (0.21)	−1.04 (6.19)	2.49 (0.17)	2.33 (0.24)	5.39 (4.44)	−3.034	0.004^***^	−0.81
Caudate	GMV	3949.34 (441.15)	3993.88 (412.88)	−0.59 (1.83)	3729.26 (419.28)	3769.53 (462.65)	−0.47 (1.95)	−0.235	0.815	-
Putamen	GMV	6038.81 (744.77)	6172.68 (687.78)	−1.38 (1.50)	5533.98 (796.54)	5557.87 (530.99)	0.35 (3.69)	-	0.007^*^	−0.61
Pallidum	GMV	2270.45 (188.53)	2158.69 (183.99)	2.53 (2.55)	2144.01 (217.29)	2115.27 (280.16)	0.79 (3.63)	-	0.019^*^	0.55

*SD, standard deviation; ROI, region of interest; LI values, laterality index values; OCD, obsessive-compulsive disorder; HC, healthy control; OFC, orbitalfrontal cortex; GMV, gray matter volume; SA, surface area; CT, cortical thickness; LGI, Local Gyrification Index. GMV and SA values are corrected for intracranial volume. GMV reported in mm^3^, SA in mm^2^, and CT in mm*.

† *Independent sample t-test of LIs between two groups. ^‡^2-tailed, significance at p = 0.05;*

* *p < 0.05;*

*** *p < 0.005*.

#### GMV

**Figure 2:**
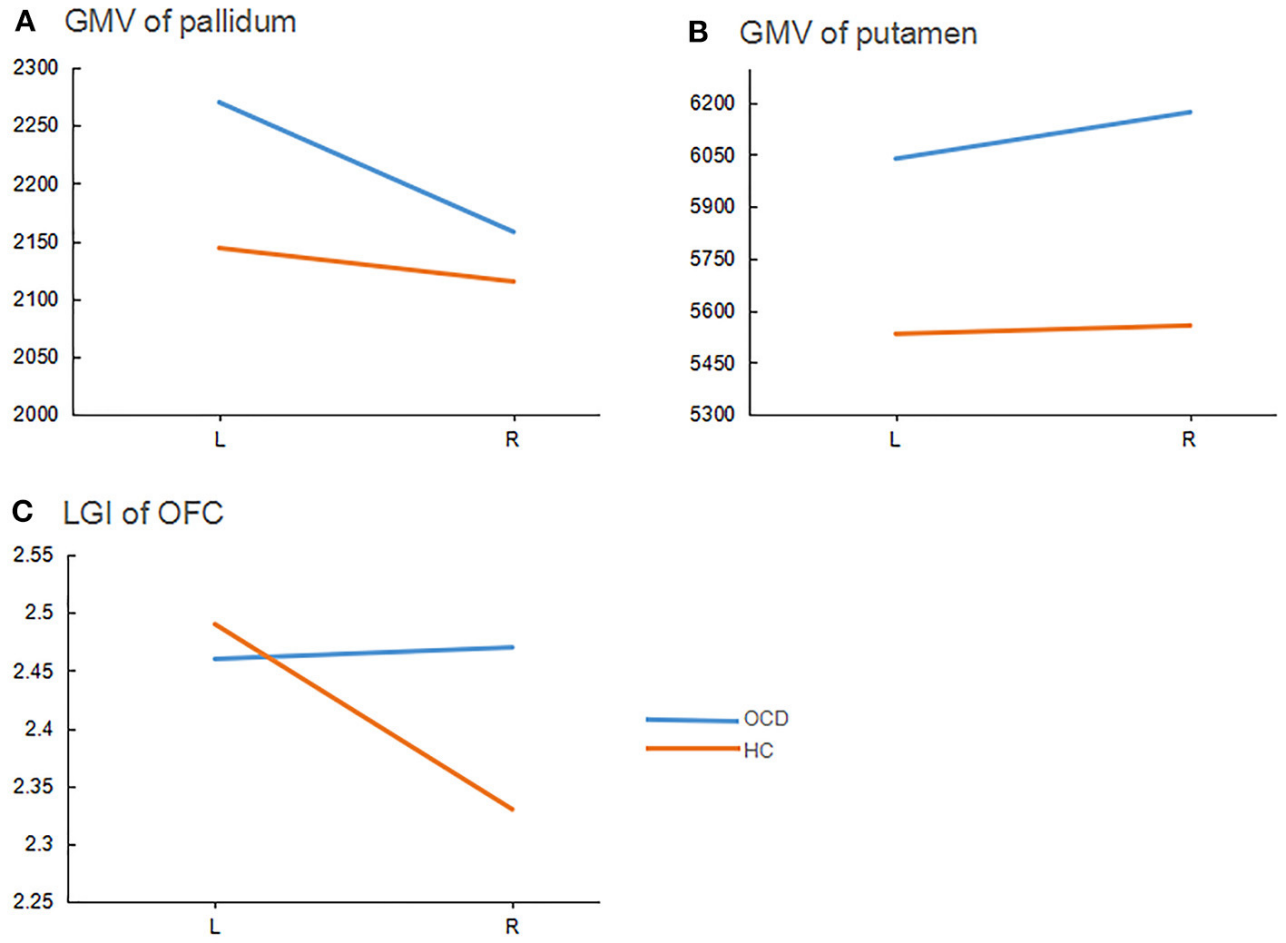
Group-hemisphere interactions of morphometric indicators for each ROI. ROI, region of interest; GMV, gray matter volume (mm^3^); OFC, orbitalfrontal cortex; L, left; R, right; LGI, local gyrification index. OCD, obsessive-compulsive disorder patients; HC, healthy controls.

For GMV of the OFC, there was no significant group difference [*F*_(1, 53)_ = 1.44, *p* = 0.24], no main effect of hemisphere [*F*_(1, 53)_ = 3.95, *p* = 0.052], and no significant group' hemisphere interaction [*F*_(1, 53)_ = 1.15, *p* = 0.29]. For GMV of the caudate nucleus, there was no significant group difference [*F*_(1, 53)_ = 1.55, *p* = 0.22], no significant main effect of hemisphere [*F*_(1, 53)_ = 0.01, *p* = 0.91], and no significant group' hemisphere interaction [*F*_(1, 53)_ = 0.007, *p* = 0.93]. For GMV of the pallidum, we observed a main effect of hemisphere [*F*_(1, 53)_ = 6.88, *p* = 0.01, η^2^ = 0.12] and a significant group' hemisphere interaction [*F*_(1, 53)_ = 6.61, *p* = 0.01, η^2^ = 0.11], but no main effect of group [*F*_(1, 53)_ = 0.33, *p* = 0.57]. Simple effect analysis showed that differences between the left and right pallidum were significant for the OCD group [*p* < 0.001, *d* = 0.60], but not for the HC group (*p* = 0.46). Relative to the HC group, the OCD group had a greater left pallidum GMV (*p* = 0.02; *d* = 0.62) but no significant difference in GMV for the right pallidum (*p* = 0.50), see Figure 2.

For the putamen, there were main effects of group [*F*_(1, 53)_ = 8.54, *p* = 0.005, η^2^ = 0.14] and hemisphere [*F*_(1, 53)_ = 20.68, *p* < 0.001, η^2^ = 0.28], as well as a significant group' hemisphere interaction [*F*_(1, 53)_ = 5.03, *p* = 0.03, η^2^ =0.09]. Simple effects analysis showed that the differences between left and right putamen were significant in the OCD group (*p* = 0.001, *d* = 0.19), but not the HC group (*p* = 0.39). The OCD group had greater GMVs in both the left putamen (*p* = 0.02; *d* = 0.65) and right putamen (*p* = 0.001; *d* =1.00) than the HC group (Figure 2).

#### SA and CT

There were no main effects on OFC SA of group [*F*_(1, 53)_ = 0.06, *p* = 0.81] or hemisphere [*F*_(1, 53)_ = 3.44, *p* = 0.07], and no significant group' hemisphere interaction [*F*_(1, 53)_ = 1.04, *p* = 0.31]. Likewise, we did not obtain significant main effects on OFC CT of group [*F*_(1, 53)_ = 2.39, *p* = 0.13] or hemisphere [*F*_(1, 53)_ = 0.37, *p* = 0.55], nor a group' hemisphere interaction [*F*_(1, 53)_ = 0.27, *p* = 0.61].

#### Gyrification

For LGI of the OFC, we obtained a main effect of hemisphere [*F*_(1, 53)_ = 4.04, *p* = 0.05, η^2^ = 0.07] and a significant group × hemisphere [*F*_(1, 53)_ = 4.37, *p* = 0.04, η^2^ =0.08], but no main effect of group [*F*_(1, 53)_ = 2.07, *p* = 0.16]. Simple effects analysis showed significant differences between the left and right OFC for the HC group (*p* = 0.007; *d* = 0.05), but not for the OCD group (*p* = 0.96). Compared to HCs, the OCD group had a larger LGI for the right OFC (*p* = 0.03; *d* = 0.69) but not for the left OFC (*p* = 0.60), see Figure 2.

### Laterality

LI of putamen and pallidum were non-normal data (*ps* < 0.05), see Supplementary Table 1. Mean LI values obtained for the OFC and the three substructures of the striatum are reported in Table 2 with their SDs and statistical values. Notably, we obtained significantly different LI values between the OCD group and HC group for GMVs of the pallidum and the putamen, as well as for LGIs of the OFC.

### Partial Correlation of LI and Psychological Variables

DEQ scores correlated negatively with LI values obtained for the LGI of the OFC, including DEQ total score (*r* = −0.34, *p* = 0.01) as well as executive memory (*r* = −0.33, *p* = 0.01), inhibition (*r* = −0.35, *p* = 0.008), positive affect (*r* = −0.27, *p* = 0.049), and negative affect (*r* = −0.26, *p* = 0.05) subscale scores. YBOCS scores correlated negatively with LI values obtained for the LGI of OFC (*r* = −0.40, *p* = 0.04).

### Effect Sizes of Group and Hemisphere Differences

**Figure 3:**
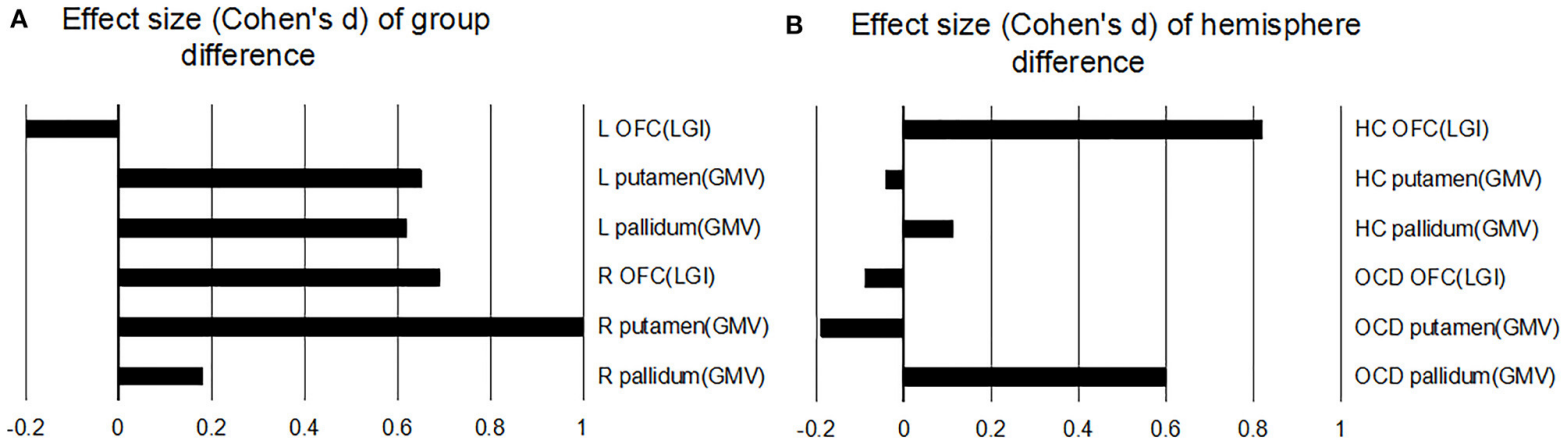
Effect sizes. Effect sizes, as represented by Cohen's d values, are shown for **(A)** group differences (OCD vs. HC) within each ROI in each hemisphere and for **(B)** hemisphere differences [left (L) vs. right (R)] in each group of each ROI. OCD, OCD patients; HC, healthy controls; GMV, gray matter volume.

Effect sizes for group differences in each ROI are reported in Figure 3 (for significant interactions), and effect sizes for differences between the left and right hemispheres within each group are presented in Figure 3. The most significant group difference was observed for GMVs of the right putamen (*d* = 1, OCD vs. HC). The most significant hemisphere difference was observed for LGIs of the OFC in the HC group (Cohen's *d* = 0.82, left vs. right).

## Discussion

In this study, we found that, relative to HCs, first-episode drug-naive young OCD patients had greater GMVs of the left pallidum and bilateral putamen, as well as an greater LGI of the right OFC, consistent with the view that OCD patients have a structurally abnormal OFC and striatum (1, 11, 12, 36). We also found that, relative to HCs, OCD patients had weaker left hemisphere LGI superiority of the OFC and GMV superiority of the putamen, as well as stronger left hemisphere GMV superiority of the pallidum. In the OCD group, LIs of OFC LGI correlated negatively with Y-BOCS scores and DEQ scores, respectively.

We found that OCD patients had a larger left pallidal GMV than HCs. A meta-analysis indicated that adult OCD patients had larger pallidal volumes than age-matched controls (1). Similarly, our study found OCD patients had larger GMVs of the bilateral putamen than HCs. Shape analysis revealed that the segmented putamen was larger than normal in OCD patients, while a voxel based morphometry study indicated that OCD patients had larger right putamen volumes than normal (1). Variability of subcortical structural alteration results in OCD may be due to different analysis methods, limited statistical power, and clinical heterogeneity with respect to patient profile and developmental stage (33).

Although OFC abnormalities have been widely reported in OCD, the results have been inconsistent (13, 17, 28), and few studies have studied cortical folding of the OFC directly. Relative to other indicators, cortical folding reflects earlier neurodevelopmental processes, and some mental disorders have been linked with abnormalities in cortical folding (58). Thus, analyzing it in OCD may reveal potential neurodevelopmental risk factors of OCD, such as CSTC loop abnormalities (37). The present findings of greater LGIs of OFC in OCD patients are consistent with the possibility that abnormal cortical folding may play an important role in the pathogenesis of OCD.

We also found that LI values obtained for the GMVs of the pallidum and putamen and for LGIs of the OFC differed between the OCD and HC groups. Hemispheric asymmetry is a basic feature of the brain that is, like cortical folding, an indicator of early neurodevelopment (59, 60). Goldberg found that heteromodal inferoparietal and lateral prefrontal cortices are more extensive in the right than in the left hemisphere, whereas heteromodal mesial and orbital prefrontal and cingulate cortices are more extensive in the left than in the right hemisphere (61). Impaired early neurodevelopment including abnormalities in brain lateralization may be etiological factors of some neurobehavioral disorders (59), such as autism spectrum disorder (62). Although the possibility that atypical lateralization may be involved in OCD, there is little direct information on the matter.

In this study, we found a significant group differences in LI values of LGIs for the OFC, wherein OCD patients had a less dominant left-side superiority than HCs, with HCs showing the typical relative enlargement of the left OFC over the right. The OFC is critical for salience-driven decision-making guided by internal states, motivations, and needs (59). The left OFC is closely related to speech and logic, whereas the right OFC has been closely related to emotional experience (59). Reduced left lateralization of the OFC in OCD patients suggests that their logical functions may be weakened while their emotional experiences may be enhanced, a supposition that fits with the OCD clinical characteristics of unnecessary repetitive behaviors and emotional distress (DSM-5, 2013).

The OCD group also had weakened left-side dominance of the putamen with respect to GMVs. The putamen controls autonomic movements. In the general population, the putamen is more dominant in the left hemisphere than in the right, and putamen injury can disrupt autonomic nervous system functions (59). Depression severity has been associated with the GMV of the left putamen (63). The bilaterally reduced putamen with weakened left dominance in OCD patients reported here may underlie, at least in part, the compulsive behavior and depression seen in OCD patients.

The pallidum, which plays an important role in regulation of body movement, typically, shows left-hemisphere dominance (59). Pathological changes affecting the pallidum may result in increased muscle tone, decreased movement, and resting tremor. Moreover, diagnoses involving a compromised the pallidum are often associated with obsessive-compulsive symptoms. Hence, the present findings of a greater GMV of the left pallidum in OCD patients and enhancement of left pallidal dominance, relative to HCs, are consistent with the repetitive behaviors characteristic of OCD and thus suggest that these alterations could underlie compulsive behaviors in OCD patients.

It is noteworthy that our LI values correlated with psychological variables, namely Y-BOCS and DEQ scores, in the OCD group. Previously, Tang found that the GMV of the left anterior insula correlated positively with Y-BOCS scores, while the GMV of the right dorsolateral prefrontal cortex correlated negatively with Y-BOCS scores (64). However, to the best of our knowledge, the relationship OFC LGI lateralization and OCD clinical characteristics had not been examined previously. The present correlation analyses indicated that OCD patients with higher Y-BOCS obsession scores tended to have less dominant left side superiority. Y-BOCS scores in OCD patients have been reported previously to correlate negatively with GMVs of the bilateral OFC (65). Our results further suggest that OCD symptom severity may be associated with abnormal lateralization LGI patterns in the OFC.

LI values of the LGI for the OFC were also inversely correlated with DEQ total and subscale scores in OCD patients. Hence, OCD patients with weaker left OFC dominance tended to exhibit greater executive dysfunction. Burgess and colleagues found that right dorsolateral prefrontal gyrus damage was associated with impairments in the ability to make plans, whereas left superior frontal gyrus damage was associated with impairments in the ability to follow plans and rules (66). Our results suggest that impaired EF in OCD patients might be related to abnormal LGI lateralization of the OFC, consistent with a neurodevelopmental etiology of OCD (33).

This study had some limitations. First, being a cross-sectional rather than a longitudinal study, it could not answer the question of whether observed asymmetries were genetically determined (innate) or consequent to the development of OCD symptoms. Follow-up studies are needed to clarify the causality direction between brain abnormalities and OCD symptoms. Second, only gray matter abnormalities and their relationships with executive dysfunction metrics were analyzed. Task-related fMRI studies may reveal specific indicators of impaired EF in OCD. Finally, the relatively small sample size may limit the generalizability of the findings. Studies with multiple comparison correction being conducted in larger sample sizes are needed to reduce the type I error and examine the repeatability of our results.

Our study found that drug-naïve adult patients with OCD indeed have abnormalities in cortical folding and lateralization patterns, which provided further evidences that OCD is an early neurodevelopmental disorder. This finding also suggested that the abnormalities of cortical folding and lateralization patterns in OFC and striatum may be biomarkers to early identify obsessive-compulsive disorder. The Follow-up studies are needed to provide more evidences.

## Data Availability Statement

The data that support the findings of this study are available from the corresponding author upon reasonable request.

## Ethics Statement

The studies involving human participants were reviewed and approved by Ethics Committee of the Second Xiangya Hospital of Central South University. The patients/participants provided their written informed consent to participate in this study.

## Author Contributions

All authors contributed to the study conception and design. Material preparation, data analyses were performed by ZB and TC. Data collection were performed by LQ, PW, YH, LZ, LF, and LJ. The first draft of the manuscript was written by ZB. TC, LQ, and ZM revised the manuscript and all authors commented on previous versions of the manuscript. All authors read and approved the final manuscript.

## Conflict of Interest

The authors declare that the research was conducted in the absence of any commercial or financial relationships that could be construed as a potential conflict of interest.
