# Supplementary material for: Gray Matter Abnormalities of Orbitofrontal Cortex and Striatum in Drug-Naïve Adult Patients With Obsessive-Compulsive Disorder
Source: Front Psychiatry. 2021 Jun 8;12:674568. doi: 10.3389/fpsyt.2021.674568 (PMC8217443; doi:10.3389/fpsyt.2021.674568)
Supplement: Supplementary file 1 [file Presentation_1.pdf]

Supplementary Material

1 Psychological assessment

The BDI, which was used to assess depression severity, consists of 21 items, each scored on a 4-point Likert scale, ranging from 0 (none) to 3 (very strong) with a total score range of 0–63 and higher scores indicating more severe depression symptoms.

The STAI, which was used to assess anxiety level, consists of 40 items, with items 1–20 and items 21–40 constituting the state (STAI-S) and trait (STAI-T) subscales, respectively. Each item is scored on a 4-point Likert scale, ranging from 1 (none) to 4 (very strong), and a score range of 20–80 for each subscale. Higher scores indicate higher anxiety levels.

The DEQ, which was used to detect executive dysfunction, consists of 20 items constituting five subscales as follows: inhibition (7 items), intentionality (5 items), executive memory (3 items), positive affect (3 items), and negative affect (2 items). The DEQ items are scored on a 5-point Likert scale, ranging from 0 (never) to 4 (very often), with a total score range of 0–80 and higher scores indicating more executive problems.

The 10-item Y-BOCS was used to assess the severity and symptom profile of obsessive-compulsive disorder. It consists of obsession (items 1–5) and compulsion (items 6–10) subscales. Each item is scored on a 5-point Likert scale, ranging from 0 (none) to 4 (very strong). The Y-BOCS score ranges are 0–20 for each subscale and 0–40 for the total score, with higher scores indicating greater severity of obsessive-compulsive disorder.

2 Calculation of Laterality Index

Laterality Index (LI) was calculated for each subject using the following equation:

$$LI = 100 \times (Left - Right) \div (Left + Right)$$

The possible LI range was -100 to +100 with positive values indicating leftward asymmetry, negative values indicating rightward asymmetry, and zero indicating perfect symmetry.

3 Normality test of LI

Shapiro-Wilk test was used to verify the normality of LI. Two sample t test were applied to examine the regional LI differences when LI was normal data, and permutation test (N=10000) were applied to examine the regional LI differences between OCD group and HC group when LI was non-normal data.

The normality test of LI were shown in Table S1. LI of putamen and pallidum were non-normal data (ps < 0.05).

Table S1 Shapiro-Wilk test for normality of LI

		group	Statistics values	df	*p*
Putamen		HC	.621	29	.000***
		OCD	.889	27	.007**
Pallidum		HC	.924	29	.038*
		OCD	.977	27	.801
Caudate		HC	.979	29	.823
		OCD	.946	27	.174
OFC	LGI	HC	.977	29	.754
		OCD	.962	27	.408
	GMV	HC	.973	29	.634
		OCD	.970	27	.590
	SA	HC	.975	29	.713
		OCD	.979	27	.838
	CT	HC	.982	29	.880
		OCD	.965	27	.481

LI, laterality index; HC, healthy control; OCD, obsessive-compulsive disorder; OFC, orbitalfrontal cortex; LGI, Local Gyrification Index; GMV, gray matter volume; SA, surface area; CT, cortical thickness; *$p < 0.05$; ** $p < 0.01$; ***$p < 0.001$.
